# Exploring Applicants' Perceptions of the Social Media Presence of Orthopedic Surgery Residency Programs

**DOI:** 10.7759/cureus.76914

**Published:** 2025-01-04

**Authors:** Samantha N Olson, Andrew J Rothka, Micah Richardson, Nikkole Haines

**Affiliations:** 1 Department of Orthopedics, Penn State College of Medicine, Hershey, USA; 2 Department of Orthopedics, Penn State Health Milton S. Hershey Medical Center, Hershey, USA

**Keywords:** instagram, orthopedics, residency, social media, stereotypes

## Abstract

Introduction

In recent years, medical students and residents across the country have begun to utilize social media as a tool to connect. With more access to technology than ever before, medical students are using residency social media pages to find out more information about the culture of residency programs. Global pandemics, such as COVID-19, assisted in precipitating these changes in resident recruitment efforts due to truncated in-person interactions and limited sub-internship rotations. Additionally, the transition to virtual interviews further pushed medical students to find new ways to connect with residency programs. In response, orthopedic residency programs increased their social media presence to share information and facilitate virtual interactions with applicants. Our goal was to determine the platforms and content most influential for applicants choosing orthopedic surgery residency programs to assess the changing landscape of social media utilization.

Methodology

Applicants to a single academic orthopedic surgery residency program from 2017 to 2022 were surveyed. Participants were asked to describe personal use of social media, encounters with residency programs on social media, and perceptions of the social media presence and content of orthopedic surgery residency programs. Surveys were distributed to participants via email including a description of the study requesting voluntary participation. Each subject received, at most, one reminder email. The data was analyzed to determine applicant perceptions of the social media presence of Orthopedic Surgery residency programs.

Results

The survey was distributed to 3690 applicants to Penn State’s Orthopedic Surgery Residency Program from the entering class of 2018-2022 in addition to the 19 Penn State fourth-year medical students applying to Orthopedics. A total of 102 people responded to the survey, with a response rate of 3.1%. Of the total number of respondents, 88.2% thought Instagram was the best platform for a residency program to use, and over 65% of respondents would use Instagram to interact with a residency program. Most respondents prefer Resident Biographies, Social Gatherings/Family Life, Day-in-the-Life, Program Culture, and Attending Biographies posted on the Instagram page every week. Applicants observed drinking, politics, and sexist language on social media pages of orthopedic surgery residency programs that were undesirable and caused a negative perception of that program.

Conclusions

Social media usage has drastically increased in recent years. Global pandemics and increased social media usage among the US population have allowed social media platforms to become a powerful tool for marketing orthopedic surgery residency programs. This survey study examined applicant perceptions of the social media presence of orthopedic surgery residency programs. Instagram was the most widely used and influential. According to surveyed participants, programs desiring to create the most positive impact through social media should aim for weekly posts highlighting the people, events, and everyday life of the orthopedic residency while avoiding controversial content that may ostracize applicants.

## Introduction

Social media is a beneficial venue for virtual education, collaboration, and networking. The use of social media has been expanded in recent years due to global events. For example, during the COVID-19 global pandemic, orthopedic surgery residency programs and applicants alike encountered unprecedented challenges. The initiation of virtual interviews and limited sub-internship rotations led to restricted opportunities for in-person evaluations. Applicants had limited opportunities to interact with programs of interest; many of them did not have time to display their interests and abilities or obtain letters of recommendation. Residency programs lost critical opportunities to demonstrate their program culture and to evaluate or recruit applicants that aligned with those goals. Since the pandemic, these virtual interviews have continued to be a popular choice for orthopedics. In a JBJS article that surveyed orthopedics program directors (PD), 65% of the PDs who responded offered virtual interviews from the 2023-2024 cycle [1]. In the 2024-2025 cycle, orthopedic surgery residencies have the option of conducting virtual or in-person interviews. These changes to the orthopedic surgery match process have created the need for new ways to connect and learn about residency programs.

Due to the new match process landscape, orthopedic surgery residency programs drastically increased their social media presence to showcase their strengths and recruit the best applicants. Between May 2019 and November 2020, social media accounts for orthopedic surgery residency programs increased by 300% [2]. Specifically, Instagram accounts for residency and fellowships increased by 620%, Twitter/X by 117%, and Facebook by 69% [3]. Among the various platforms, Instagram had the most growth and usage, followed by Twitter and Facebook [4]. Previous literature has highlighted this rise in social media accounts and content, but an analysis of the applicant perspective or response to programs' social media presence has not been well described.

This boom in social media usage has altered the ways that applicants and residencies interact. Applicants are utilizing the residency social media hubs to get a newfound look into the different programs [5]. Students are no longer confined to away rotations and interviews to create opinions about residency programs. After 2020, 115 (57.2%) residency programs had joined Instagram, with 101 (87.8%) of these programs being managed by current residents [6]. Over 75% of these Instagram accounts were made after March 1, 2020 [6]. As a field, orthopedic surgery has adapted to these new technological advances to establish connections and share knowledge with the next generation [3]. In a recently published systematic review of social media usage by orthopedic surgery residency programs, it was found that social media platforms are being utilized as both a recruitment tool and a way to provide information to residency applicants [3].

The goal of our study was to survey orthopedic surgery applicants to determine their perceptions and preferences regarding social media content distributed by orthopedic surgery programs. In this study, we explored the ways that social media can be utilized for the benefit of residency programs and applicants alike. We hypothesized that applicants would favor programs utilizing social media to reach a broader audience, particularly those using Instagram. This aligns with previous literature demonstrating Instagram to be the most popular medium used by residency programs. In addition, we hypothesize that respondents prefer to see all aspects of a residency program, including day-in-the-life information, research, and personalized stories of residents published on social media to reflect the culture of the program.

The information presented in this paper was presented as a poster at the Pennsylvania Orthopedic Society meeting in Pittsburgh, Pennsylvania, in September of 2023.

## Materials and methods

We conducted a single-center study approved by the Penn State College of Medicine Internal Review Board with the study titled STUDY00019739 on April 21, 2022. Consent was implied by survey participation. An individual REDCap survey link was emailed to individuals who previously graduated from medical school and applied to the orthopedic residency at Penn State Health Milton S. Hershey Medical Center (PSHMC) between 2017 and 2022 as well as to Penn State medical students expected to graduate in 2023 (Appendix). The survey was sent out between July 25, 2022, and August 12, 2022. The editing, manuscript writing, and presentations took place from April 21, 2022, through October 22, 2024. 

Study subjects

Data were collected from current medical students and residents in orthopedics. The purpose was to determine if there was a difference in residency applicant opinions regarding residency social media presence following the recent surge in social media accounts following the global pandemic. Only participants living in the United States were included. All responses were de-identified during the analysis process for the survey to remain anonymous. Protected Health Information (PHI) was neither used nor disclosed during the survey. The number of current students polled was low, but the survey was distributed to many individuals who had previously applied to the orthopedic surgery residency program at Penn State. 

An anonymous survey on REDCap was utilized to collect and store data. Individuals who previously applied to the orthopedic surgery residency program at Penn State were sent a unique survey link to the email that they provided during their application. The current medical students were contacted through their class email list. Each participant received an initial email invitation to participate in the survey. A reminder was sent after two weeks to those who had not responded to the survey. The survey remained open for a total of four weeks. Emails were not connected to the responses of the survey, but individuals who completed the survey were marked as complete in the REDCap system. The survey included a combination of single-answer, multiple-select, and open-response questions.

Branching logic was used for some questions. For example, only if participants selected *Yes* to the question *Do you have your own social media account(s)?* were they given a list of platforms to choose from. This prevented individuals from answering non-applicable survey questions, making the survey less onerous and more efficient. 

Participants who completed the survey were invited to enter their email into a drawing for a $50 Amazon gift card. A link to another REDCap survey was provided at the end of the questionnaire. This survey led to a new dataset within REDCap, keeping responses separate from the emails provided for the drawing. The funding for this gift card came from the gift fund of a corresponding author through the Department of Orthopedic Surgery at PSHMC.

Statistical methods

Our study was predominantly descriptive. We quantified responses and determined percentages of respondent preferences to determine applicant characteristics and their perceptions of different aspects of social media content. For comparisons, a chi-square test was used to determine the *P*-value.

## Results

The survey was distributed via email to 3,690 prior applicants to the residency program at Penn State and to the 19 members of the class of 2023 who were applying to orthopedics. A total of 440 email addresses were no longer in use and were unable to receive the survey, leaving a total population of 3,269. A total of 102 people responded, resulting in a response rate of 3.1% (102 out of 3,269). Only respondents with completed surveys were included in the data. Because of the branching logic used for some questions, there was a different number of responses for various questions.

Demographic information of the survey respondents

Respondents were asked background questions to determine the demographics of survey respondents. Table 1 summarizes information regarding age, gender, sexuality, race/ethnicity, and marital status. Of the 102 respondents, 95 (93.14%) were between the ages of 21 and 34, and 7 (6.86%) were above the age of 35. Of the 102 respondents, 80 (78.43%) were identified as male, and 22 (21.57%) were identified as female. Regarding sexuality, of 102, 96 (94.12%) were heterosexual, 2 (1.96%) were homosexual, and 4 (3.92%) were bisexual. Respondents were asked to self-identify their race/ethnicity, and 86 of 102 (84.21%) were White or Caucasian individuals. Of the 102 respondents, 58 (56.86%) were married, and 43 (42.16%) were single (never married). 

**Table 1:** Demographic information for survey respondents.

Demographic	Category	Number of respondents	Percentage of responses
Age (years)	21-34	95	93.1%
	≥35	7	6.9%
Gender	Male	80	78.4%
	Female	22	21.6
Sexuality	Heterosexual	96	94.1%
	Homosexual	2	2.0%
	Bisexual	4	3.9%
Race/Ethnicity	Asian or Pacific Islander	6	5.8%
	Black or African American	2	2.0%
	Hispanic or Latino	4	3.9%
	White or Caucasian	86	84.3%
	Multiracial or Biracial	2	2.0%
	A race/ethnicity not listed	2	2.0%
Marital status	Single (never married)	43	42.1%
	Married	58	56.9%
	Divorced	1	1.0%

Survey respondents were from all over the United States. When asked what state in which the respondent attended medical school, individuals responded with 28 different states. When asked in which state the respondent is located for residency, there were 25 different states represented. For both questions, the three most common states were Pennsylvania, Texas, and New York.

Residents from all years of residency were represented in the survey. Of the total respondents, 81.4% did not or did not plan to take time off between medical school and residency, whereas 18.6% of respondents did or did plan to take time off before starting residency. Most respondents (84.31%) applied for residency one time.

Participant’s medical school graduation year 

**Figure 1:**
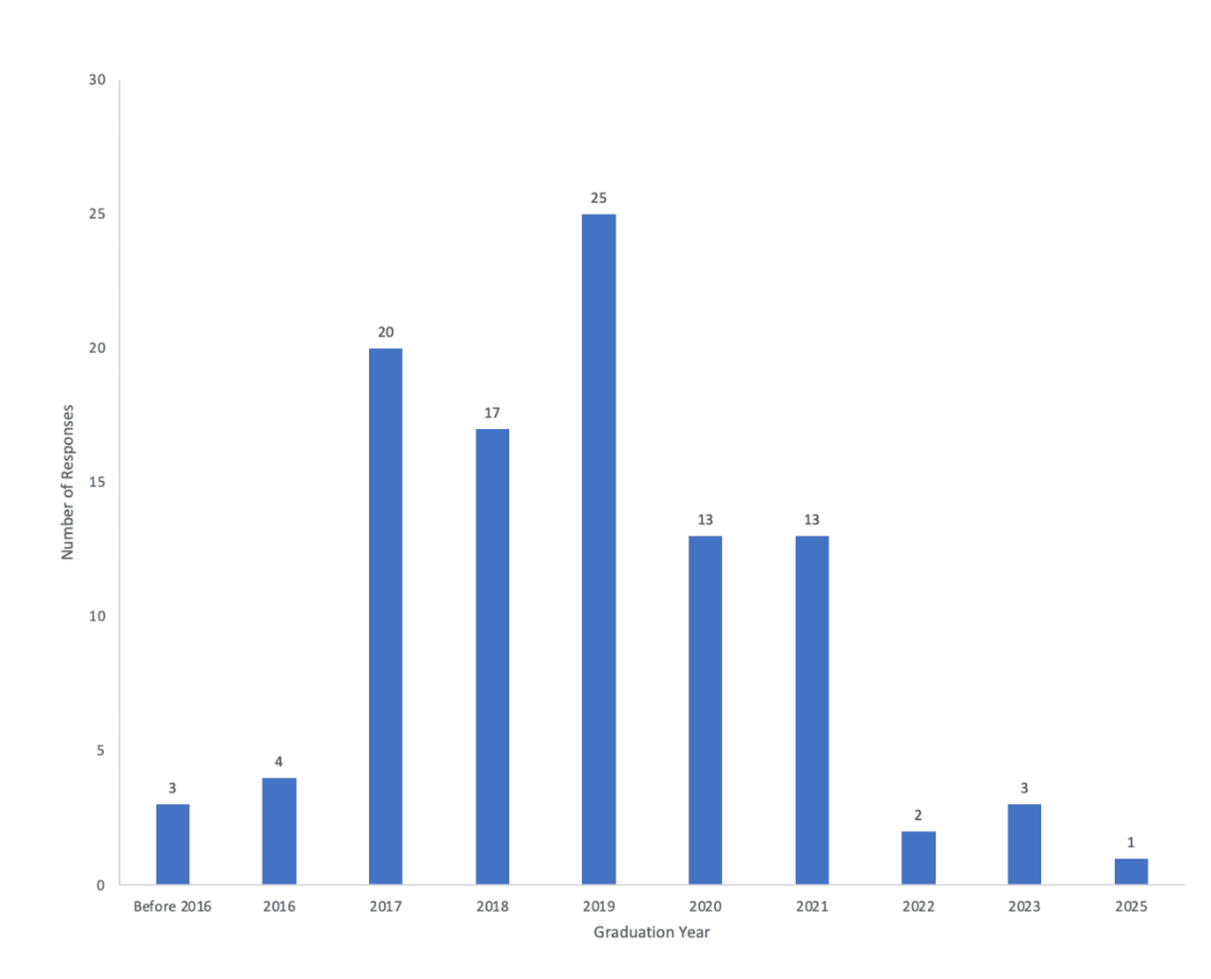
Participant’s medical school graduation year. Most individuals graduated from medical school between 2017 and 2019.

Participants of the survey were asked what year they graduated from medical school or their expected year of graduation from medical school (Figure 1). The responses ranged from 2010 to 2025. The few responses that were before 2016 were combined into one smaller data point for the figure. Most respondents of the survey graduated from medical school in 2017, 2018, or 2019.

Appealing content for an orthopedic surgery residency social media 

**Figure 2:**
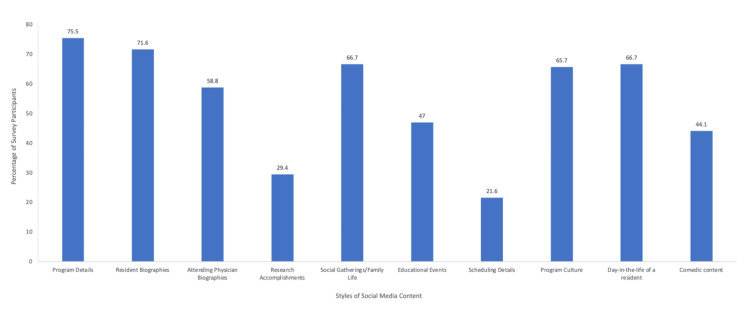
Appealing content for orthopedic surgery residency social media. Responses support presenting a wide variety of content on orthopedic surgery residency social media pages, with program details such as typical cases, volume, travel time, attending participation, and autonomy having the highest number of responses.

Respondents were asked to check all boxes for topics they found appealing. The highest percentage of responses were for program details such as typical cases, volumes, travel time, attending participation, autonomy, and program size (75.5%), followed by resident biographies (71.6%), social gatherings/family life (66.7%), and a day in the life of a resident (66.7%) (Figure 2). The lowest number of responses preferred information on scheduling, but over 20% of the cohort of the study expressed interest in learning about scheduling through social media platforms. 

Preferred post frequency of an orthopedic surgery residency program 

**Figure 3:**
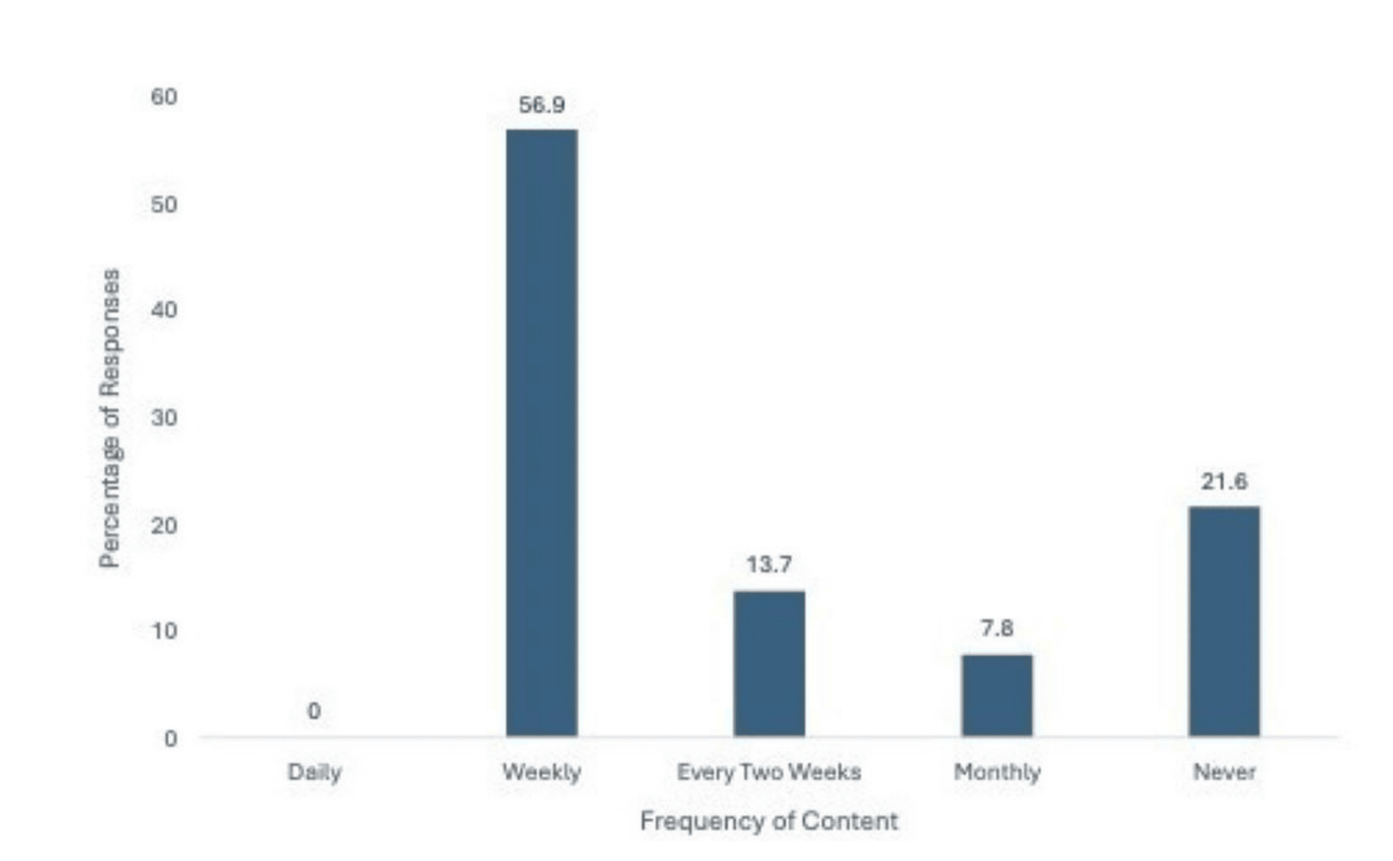
Preferred post frequency of an orthopedic surgery residency program. Most respondents (56.9%) preferred weekly frequency for residency program social media posts.

The survey found that the preferred social media post frequency was weekly (56.9%; Figure 3). The second highest cohort wished to never see a post from a residency program (21.6%). Of the total respondents, 13.7% preferred biweekly posts, whereas only 7.8% preferred monthly posts. No respondents desired to see daily posts from an orthopedic surgery residency program on social media.

Personal social media usage

**Figure 4:**
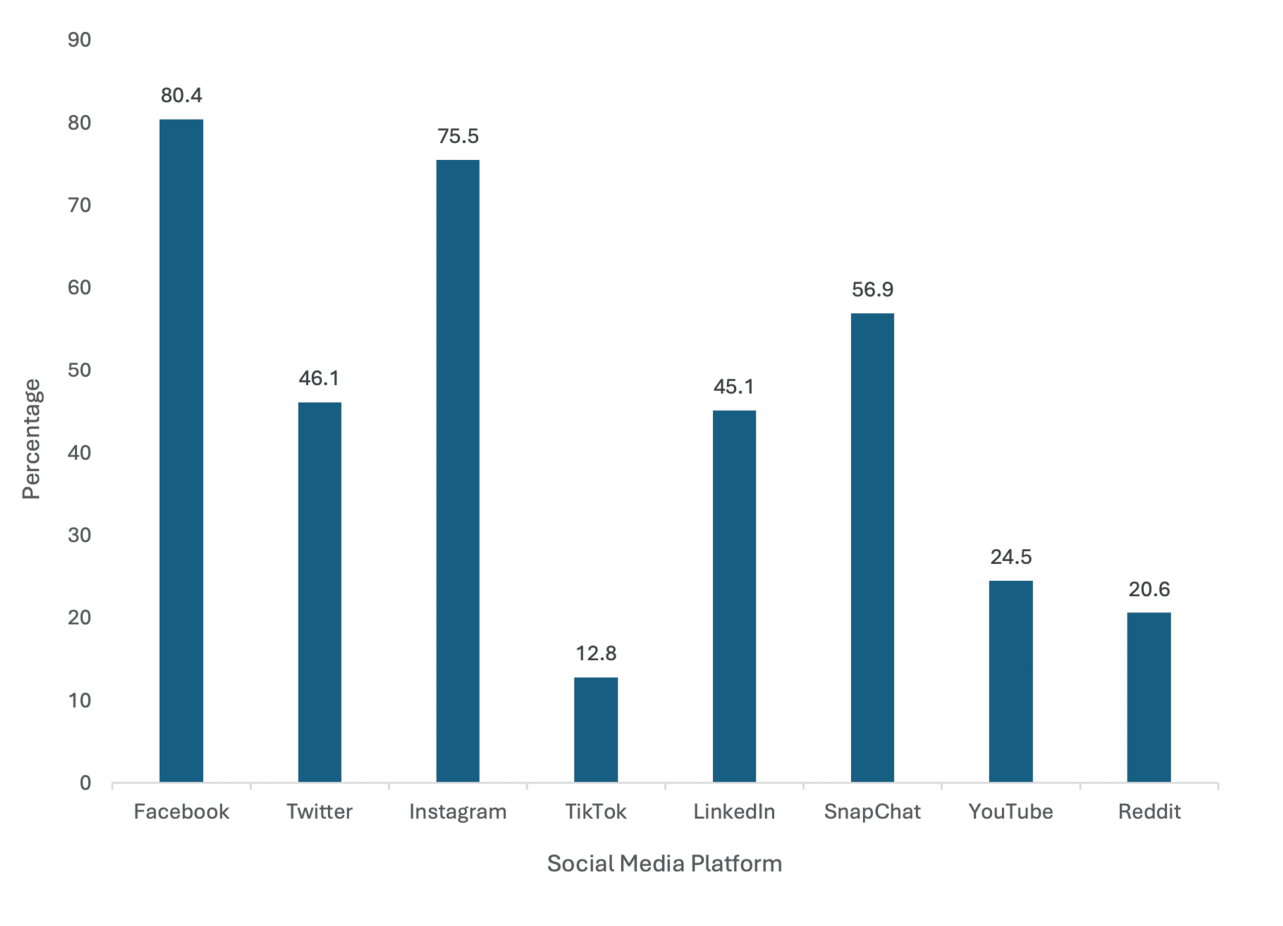
Personal Social Media Usage Most respondents reported Facebook (80.4%) and Instagram (75.5%) for personal use.

Respondents to the survey were asked about personal usage of social media accounts (Figure 4). Most reported using Facebook (80.4%) and Instagram (75.5%) for personal use. Snapchat was the next most popular platform, with 56.8% of participants reporting using the application. Of the total respondents, 46.1% reported having Twitter for personal use, and 45.1% of respondents used LinkedIn. The three least popular sites were YouTube (24.5%), Reddit (20.6%), and TikTok (12.8%). Although respondents use a wide variety of social media platforms, these are not necessarily the preferred methods for professional interaction with residency programs.

Residency applicant social media engagement

**Figure 5:**
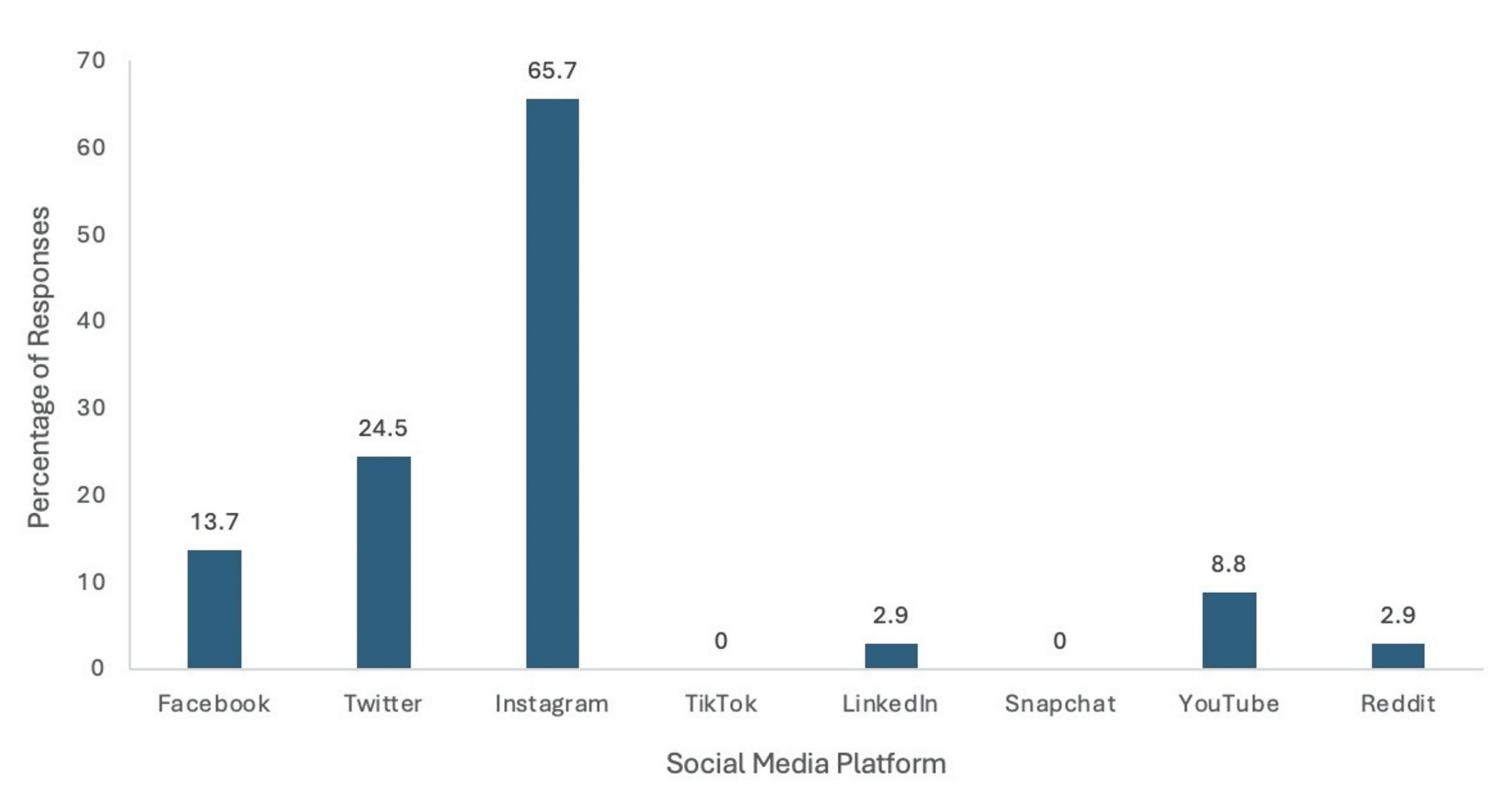
Residency applicant social media engagement. Instagram was the most utilized social media platform by applicants to interact with residency programs.

Respondents to the survey were asked to describe what platform, if any, they used to contact an orthopedic surgery residency program (Figure 5). Most respondents utilized Instagram (65.7%) to engage with a residency program. The second-most common platform was Twitter (24.5%). Of the total respondents, 13.7% used Facebook, 8.8% used YouTube, 2.9% used Reddit, and 2.9% used LinkedIn. No respondents noted using TikTok or Snapchat to engage with a residency program.

Most effective residency social media platforms 

**Figure 6:**
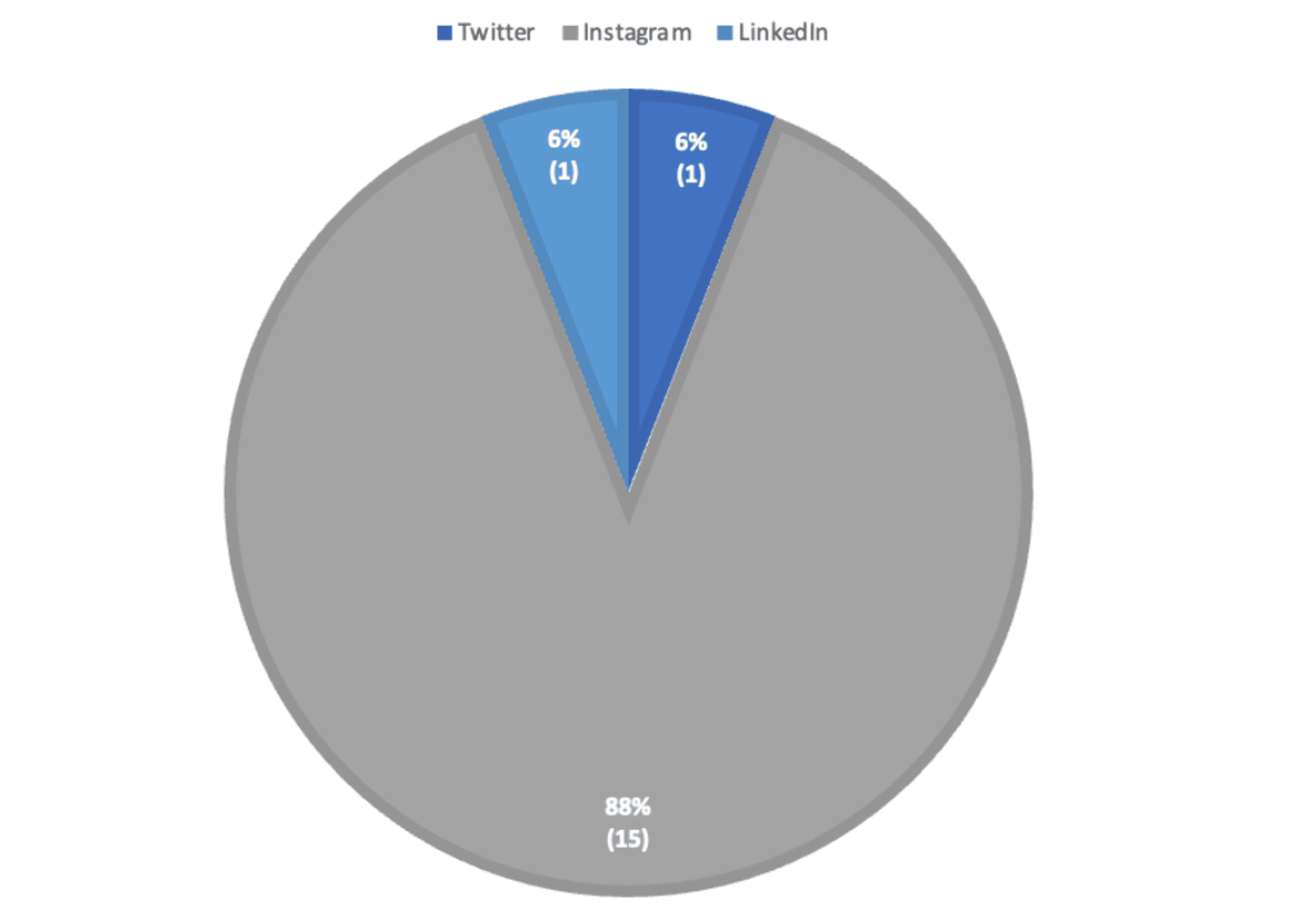
Most effective residency social media platform. Instagram was the most effective social media platform for attracting potential applicants (88.2%). The *n* values for each survey response are represented in parentheses below the percentages.

Participants were asked to identify the social media platform that was most effective at interesting them in an orthopedic surgery residency program (Figure 6). An overwhelming majority of respondents selected Instagram (88.2%) as the most effective social media platform. A minority of participants also selected Facebook and LinkedIn as the most effective, with each platform receiving recognition from 5.8% of respondents.

Social media platforms utilized the most to interact with residency programs 

**Figure 7:**
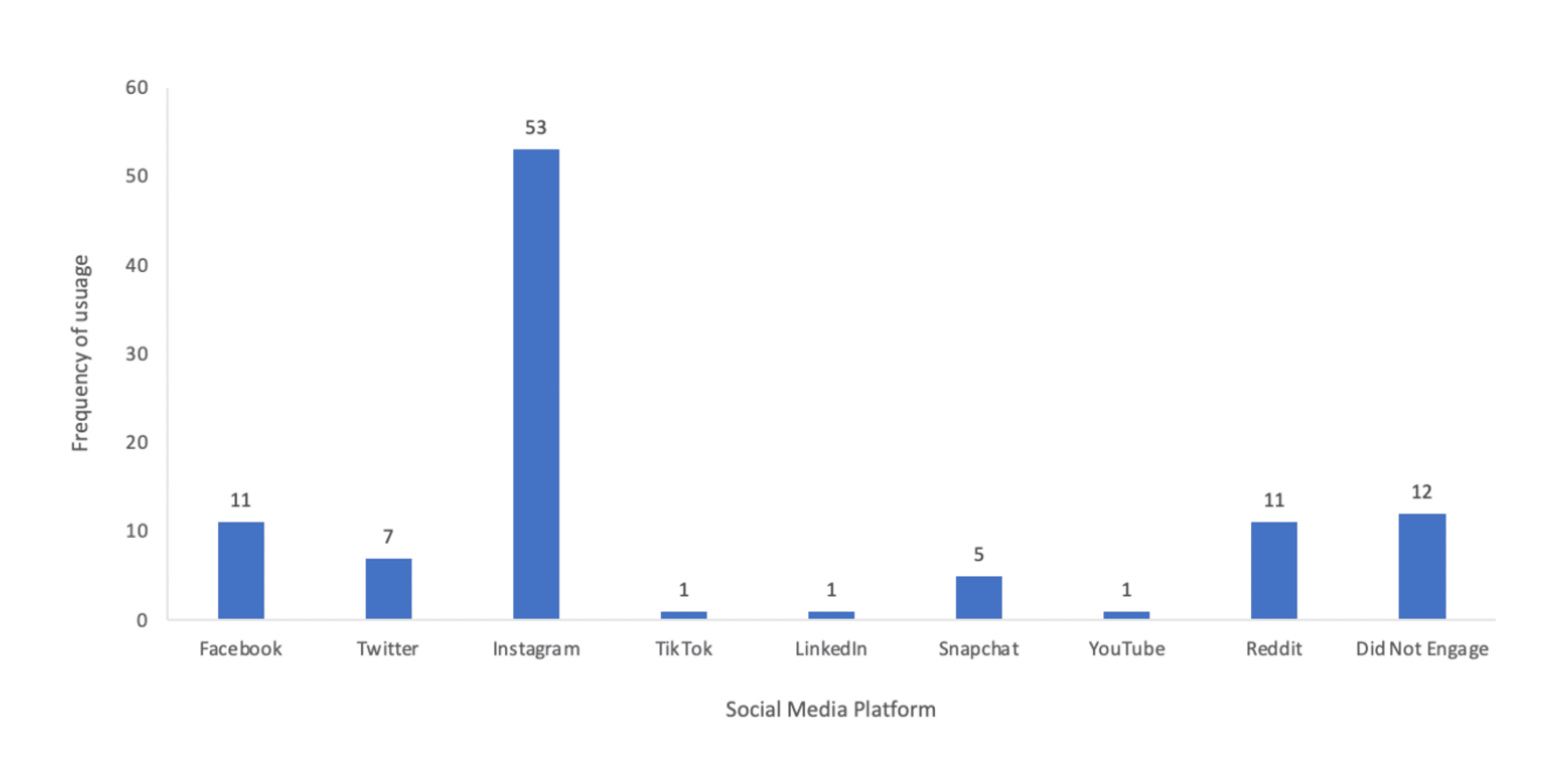
Social media platform utilized the most to interact with residency programs. Instagram was the social media platform where participants spent the most time engaging with content, with 53 respondents stating that they used Instagram the most (52%).

Most survey participants reported using Instagram the most to interact with an orthopedic surgery residency program (52%) (Figure 7). The second most frequent response was that the individual did not use social media (11.8%). Of the total respondents, 10.8% reported using Facebook most to interact with a program, and another 10.8% reported using Reddit to interact with a program. Of the total respondents, 6.9% used Twitter most frequently to engage with a residency program. One percent of respondents used each of the following platforms the most: TikTok, LinkedIn, and YouTube.

Comparison of personal social media usage to professional social media usage 

**Figure 8:**
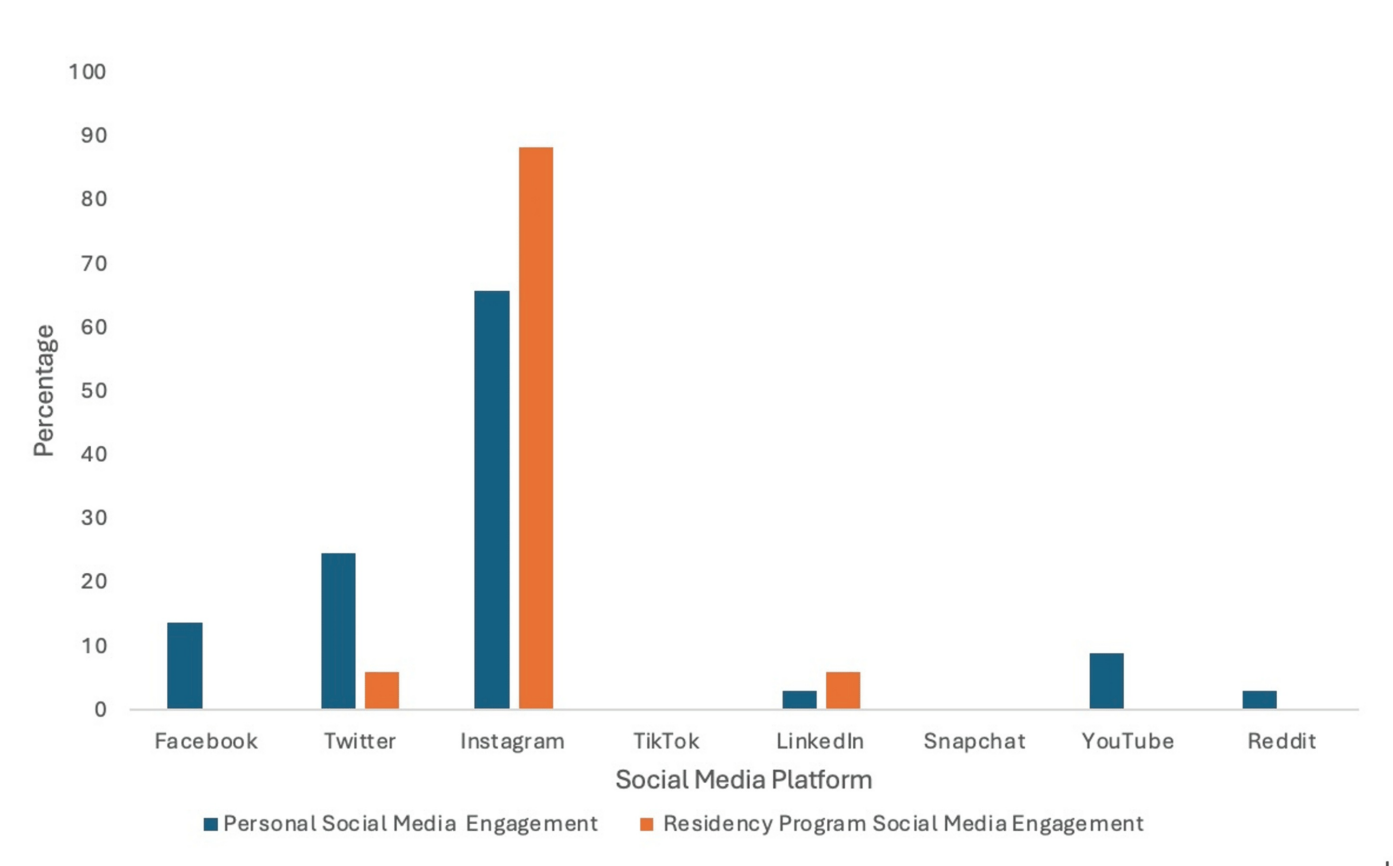
Comparison of personal social media usage to professional social media usage. Comparison of how people used social media for personal use versus for interactions with residency programs. Instagram was the most common platform for both personal and professional use.

Instagram was the most commonly utilized social media platform for both personal (65.7%) and professional (88.2%) usage (Figure 8). Twitter and LinkedIn tied for residency program social media engagement with 5.9%. Twitter was the second most common personal social media site that our survey participants engaged with. Facebook was the third most popular social media platform for personal engagement with 13.7%, and YouTube was the fourth most popular option with 8.8%. Both LinkedIn and Reddit had 2.9% engagement on personal social media accounts.

Importance of social media presence for residency programs 

**Figure 9:**
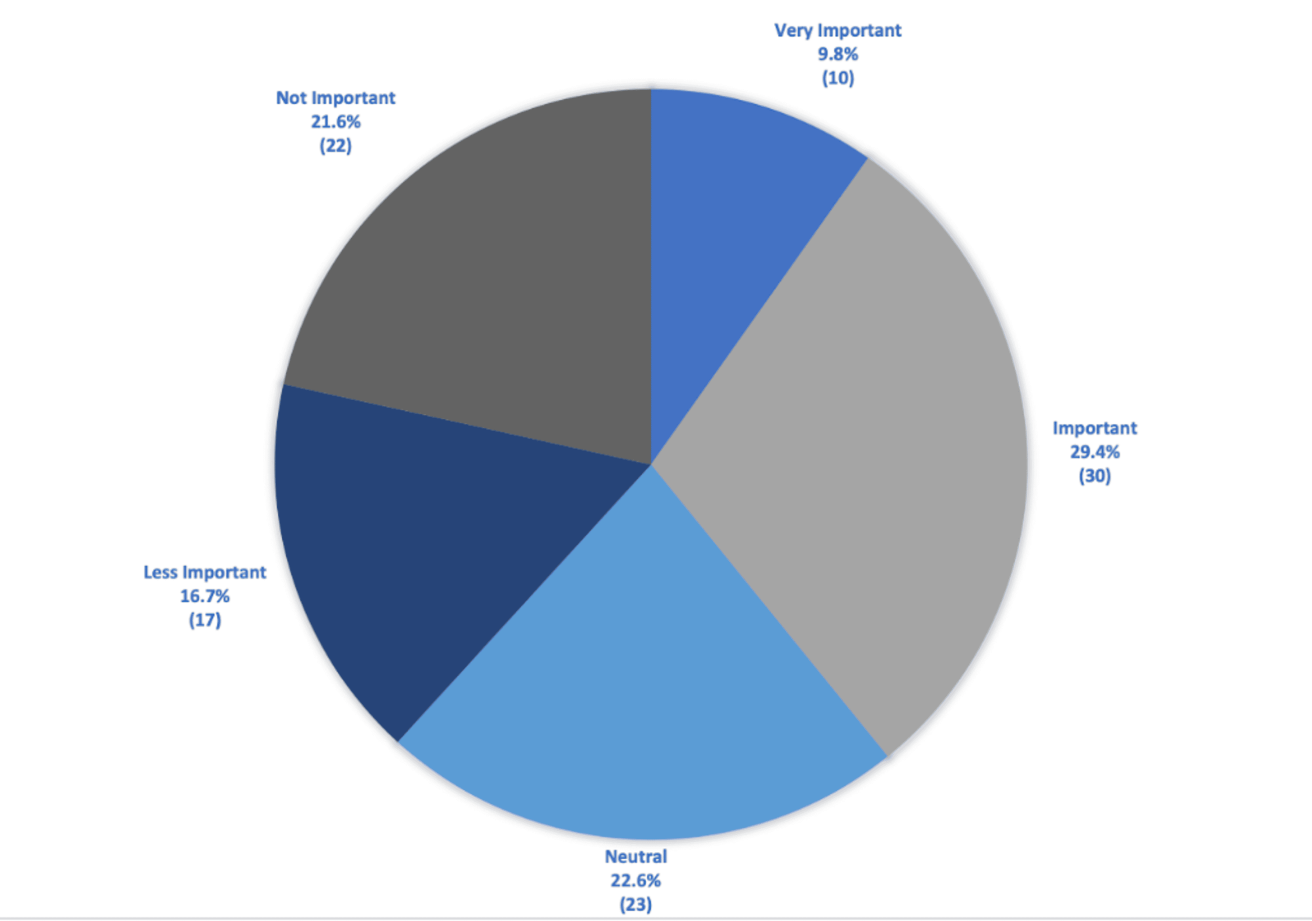
The importance of a social media presence for residency programs. A slight majority (29%) felt it was important for residency programs to have a presence on social media. The *N* values for each survey response are represented in parentheses below the percentages.

When asked about the importance of a social media presence for orthopedic surgery residency programs, the results were nearly an even split (Figure 9). Of the total respondents, 9.8% believed it was very important, 29.4% thought it was important, 22.6% had a neutral opinion, 16.7% thought it was less important, and 21.6% thought it was not important.

Engagement with orthopedic residency social media 

**Figure 10:**
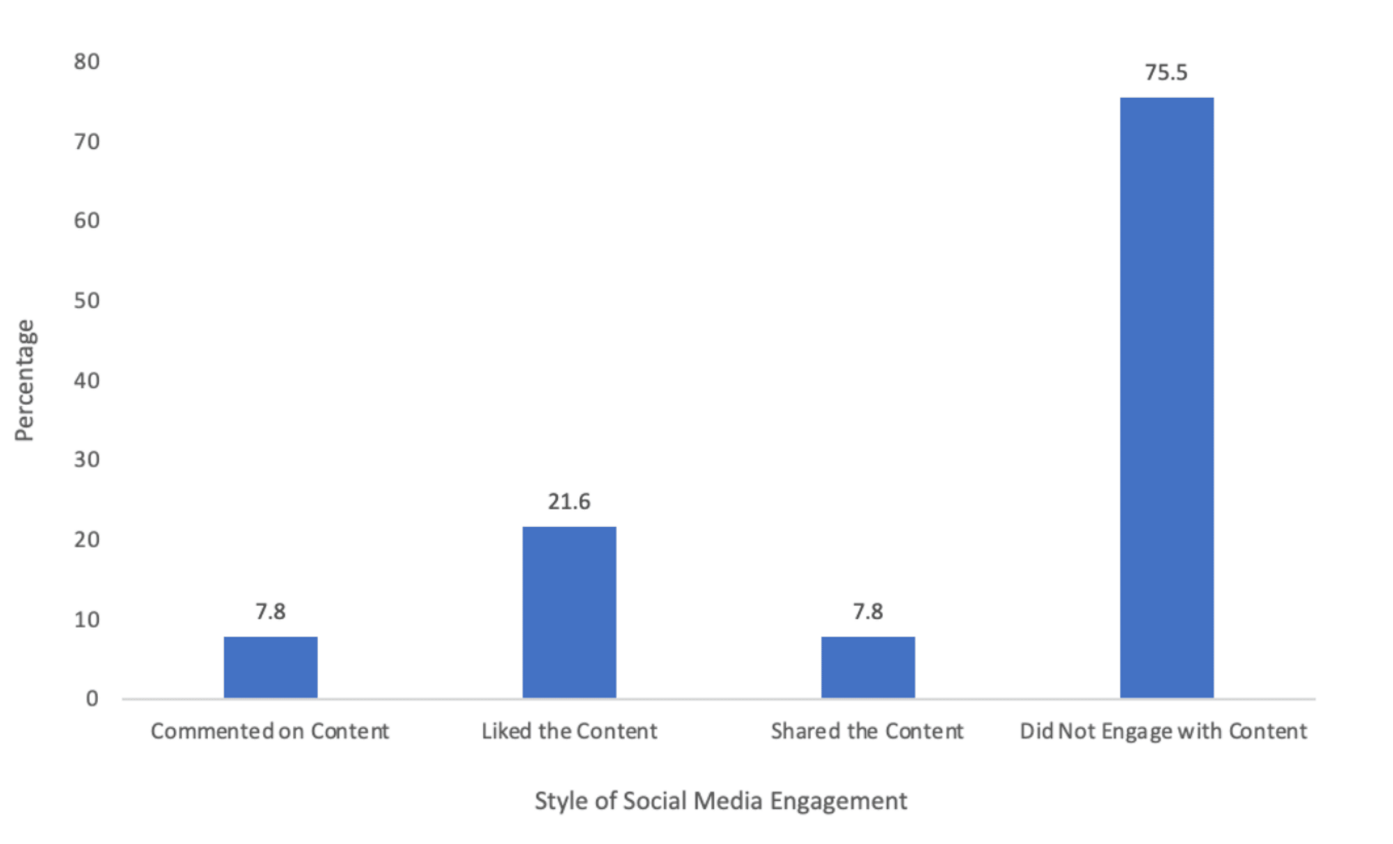
Engagement with orthopedic residency social media.

The percentage of respondents who interacted with orthopedic surgery social media accounts is presented with the action that was taken with the content (Figure 10). Of the total respondents, 21.6% liked the content shared on an orthopedic surgery residency page, 7.8% of respondents shared the content, and 7.8% commented on the content. Of the survey respondents, 75.5% did not engage with orthopedic social media accounts.

Observed stereotypes related to the field of orthopedic surgery

**Figure 11:**
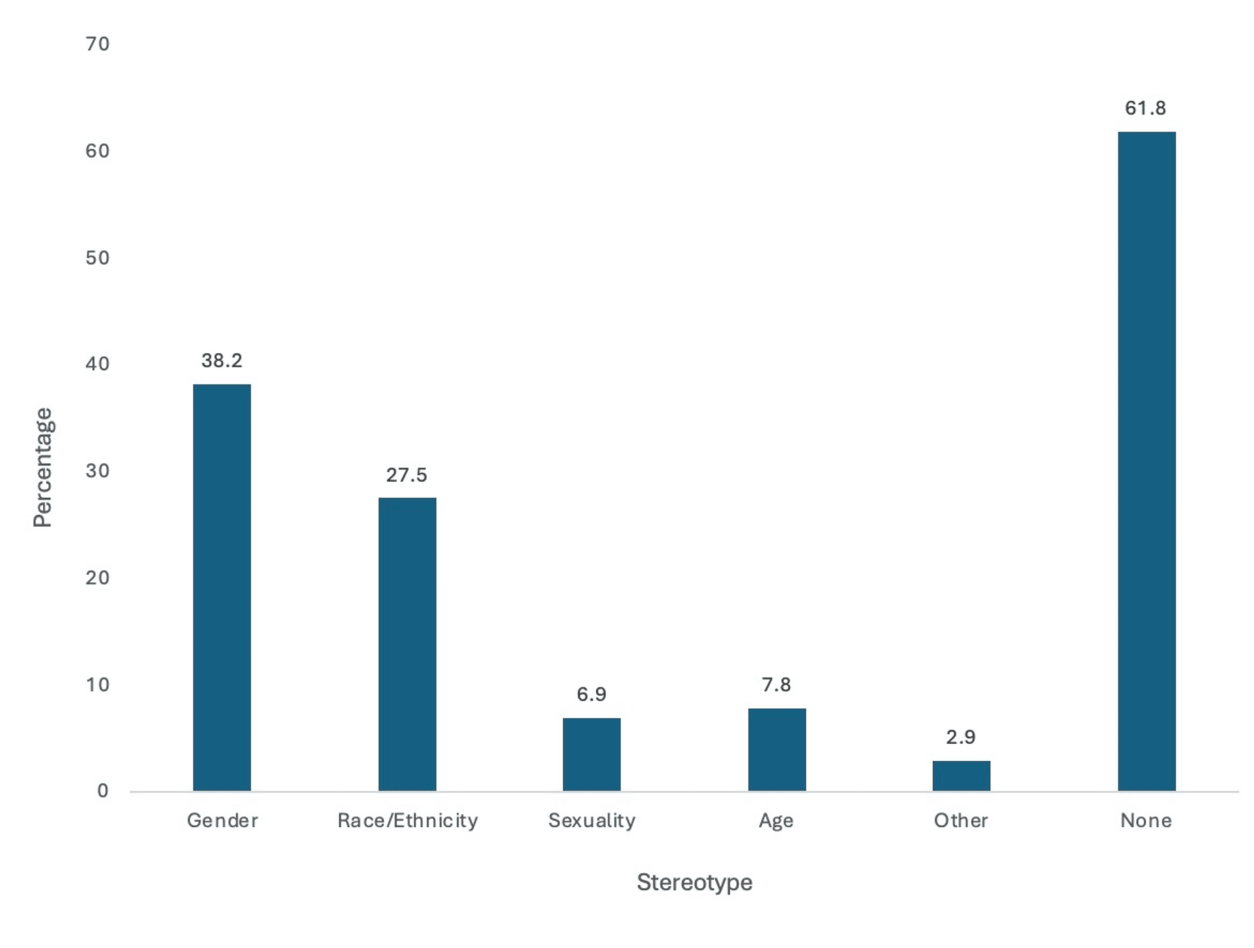
Observed stereotypes related to orthopedic surgery. Orthopedic applicants observed gender, race, ethnicity, sexuality, and age stereotypes on orthopedic-focused social media platforms.

Stereotypes regarding orthopedic social media activity were observed by survey participants and recorded (Figure 11). While the majority (61.8%) did not see stereotypes on social media related to orthopedic surgery, 38.2% viewed stereotypes related to sex. Of the survey participants, 27.5% viewed stereotypes about race/ethnicity, 7.8% viewed stereotypes based on age, 6.9% viewed stereotypes on sexuality, and 2.9% viewed stereotypes on other topics on orthopedic social media. Of the survey participants, 72.7% of females observed stereotypes on social media based on gender, and 28.7% of males reported stereotypes on social media based on gender with a *P *< 0.001. Additionally, 59.1% of females viewed stereotypes on social media based on race, and 18.8% of males viewed stereotypes on social media based on race with a *P *< 0.001. 

Evidence from social media that encouraged participants to pursue orthopedics 

When asked about the influence social media had on choosing a residency program, most people reported social media as having no effect (Table 2). A few individuals said that social media encouraged their pursuit of orthopedics. No one reported social media as discouraging them from exploring orthopedics as a specialty.

**Table 2:** Evidence from social media that encouraged participants to pursue orthopedics.

Survey responses about residency program social media accounts
Allows you to evaluate different surgical techniques, procedures, communities, etc.
Educational accounts
Found a community
Interesting accounts, journal accounts
Programs that were professional and aligned with my residency goals were sought after
Seeing strong women in orthopedic surgery
Social media is a great way to demonstrate the program’s positive aspects including educational opportunities, current residents and faculty, new research, and social life.
Good to see the program's culture
OrthoTwitter has great educational opportunities. A lot of people post pre-op and post-op X-rays as a way to learn/teach.
Seeing females in ortho

## Discussion

With increasing social media representation in the medical community, it is imperative to understand the impact of social media presence and its influence on the residency applicant. Orthopedic surgery is one of the most competitive medical specialties, making it increasingly important for residency programs to understand how to utilize and expand their social media presence to attract strong applicants [7,8]. A previous study found that orthopedic surgeons’ perceptions and opinions of institutions were positively biased based on marketing and branding, social media presence, or a combination of the two strategies [7]. Our study found that residency programs use Instagram more than other social media sites [7,9,10]. Since 2017, orthopedic surgery Instagram accounts have been growing exponentially, leading to more access for applicants to information about residency programs [8]. According to our surveyed applicants, Instagram was the most popular and influential platform. Over 50% of all orthopedic surgery residency programs in the country have Instagram accounts, and 85% of those accounts were made following the global pandemic [4,6]. With established Instagram accounts, orthopedic surgery programs can be confident that they effectively reach their target audience.

Our demographics consisted largely of heterosexual White males aged between 21 and 34 years. The study was relatively split with the number of participants who were married versus those who were single (Table 1). These results largely correlate with current demographics in orthopedic surgery programs [11]. However, we also had representation of many different races, ethnicities, and sexualities. We received responses from across the United States; 28 different states for medical school and 25 different states for residency were represented.

Most respondents graduated medical school in 2017, 2018, or 2019 and were residents when the pandemic first hit. At that time, social media was a predominant manner for staying connected to people. The social media presence of residency programs dramatically increased during this time, which allowed residency programs to maintain connections with people during an isolating time [4,6].

The focus of previous articles included general benefits and drawbacks regarding social media usage among orthopedic surgeons. A recently published study reached out to attendings, residents, medical students, and fellows to inquire about perceptions and usage of social media. They found that a professional social media account can help connect with orthopedic residency programs, but respondents indicated hesitancy regarding professionalism and best practices for interacting on social media platforms [12]. Other articles have explored etiquette and created guidelines for the creation of residency social media sites [13]. Bhayani et al. mentioned that social media accounts should share the culture of the program, be intentional and proactive about diversity, equity, and inclusion, and show the *day in the life* of resident posts [13].

Previous studies exploring the social media growth of residency programs and recommended content did not solicit opinions of what consumers would want to see on these platforms for orthopedic surgery residencies. Based on the results of our study, most people wanted to see program details, resident biographies, social gatherings/family life, and days in the life of a resident (Figure 3). These features would educate social media users about the residency program and the character of the individuals involved with the program. Instagram has been found to post more personal content, and the platform includes more content engagement features than other social media sites like X. This further confirms the importance of an Instagram presence for orthopedic residency programs [9]. Since many interviews are held online, social media can be an effective tool for individuals to learn about the program and people without ever setting foot in the hospital.

Most (56.9%) survey participants prefer to see content from a residency program every week. According to respondents, this seems to be the sweet spot to showcase program information without overburdening followers on social media. This aligns with previous studies showing that a majority, up to 98%, of medical students surveyed had some form of social media [12,14].

The perceived importance of a social media presence for a residency program was divided. 39% of respondents felt it was very important or important, 22% were neutral, and 37% felt it was less important or not important (Figure 9). This aligns with current literature that medical students are using social media to influence their perspective on orthopedic residency programs. A cross-sectional survey of residency applicants and program directors found that 69.2% of the survey participants learned about residency programs through Instagram accounts, and 34% indicated that social media influenced their rank list [15]. Another study confirmed these findings and found that programs with a social media presence had higher average rankings, showing that social media can be an important tool for residency programs to connect to applicants [16]. This is further confirmed by older studies of other surgical specialties. Plastic surgery applicants felt that residency programs' social media accounts influenced their perspective of the program and their rank list [17]. While virtual interviews saved money and limited in-person contact to prevent the spread of communicable diseases, they do not allow orthopedic surgery programs to fully elucidate the culture of their institution. Social media can help bridge this gap by providing transparent information about the program to potential applicants.

An interesting finding from our study was that women were more likely than men to observe stereotypes about orthopedic surgery, particularly those based on race and gender. Orthopedics remains a largely male-dominated field. Compared to other surgical specialties, orthopedics has the lowest percentage of women surgeons as of 2024 [18]. Additionally, the number of women in orthopedic residency programs has consistently hovered around 13.6% for several years [19]. Women may be seeing more posts about stereotypes, or men may not be picking up on the same undertones as women. In 2023, a study found that 27% of their female respondents changed their name on their social media to de-identify themselves, and 11% of female respondents deleted their social media during the application cycle due to concerns about professional content [13]. A study published in 2023 found that Instagram can be a platform that promotes women in surgery, but it lacked information on how women perceive orthopedic content on Instagram [20]. Further studies can analyze the differences in media and the perceptions of that media between men and women.

Although social media websites can create boundless connections among individuals worldwide, they can also create divisions by spreading biased, false, or otherwise negative content. When asked about stereotypes related to orthopedics on social media, a majority of respondents (63%) never observed any. Nevertheless, the two most common observed stereotypes were regarding gender and race/ethnicity (Figure 11). Despite the occasional presence of biased content, social media can strengthen connections between people [21]. We asked participants to share what they had seen on social media that encouraged them to pursue orthopedics as a specialty. Some answers included “seeing strong women in orthopedic surgery” and “programs that were professional and aligned with my residency goals.” This highlights the positives seen online about the specialty, and it provides examples of how programs can expand their reach to broader audiences (Table 2).

Based on the results of our study, our recommendation is to utilize Instagram with a post frequency of once per week in order to reach more potential applicants. Posting content such as program details and resident biographies can help to promote the residency program. Avoiding controversial or inappropriate content is critical to maintaining a professional social media presence.

There are several strengths and limitations to our study. One strength is the paucity of data regarding applicant perception of residency program-associated social media accounts. Our study provides feedback for residency programs on how to best attract applicants to their programs. Future studies should aim to receive a higher response rate to increase the reliability and generalizability of results. Future studies can also query the differences in perceptions of media between men and women given that we found that women are more likely to see stereotypes about orthopedic surgery perpetuated on social media. To our knowledge, this is the largest collection of data from surveyed orthopedic surgery residency applicants, and it provides invaluable information for orthopedic surgery residency programs seeking to connect with applicants through social media.

Limitations

Limitations were largely related to the study design. Our main obstacle was the response rate, which was only 3.1%. This introduced a high risk for nonresponse bias. Outdated emails were a barrier to reaching more participants. Emails from those who applied to the orthopedic surgery residency program at Penn State from the entering classes of 2017-2022 were polled. Since many of these applicants used school emails, there is a high chance that we had email addresses that were no longer in use. Additionally, potential sampling bias in surveying single institution applicants to an institution with an Instagram page may have influenced our results.

## Conclusions

In recent years, the global pandemic has led to an increase in social media usage and virtual interviews; these changes have catalyzed orthopedic surgery residency programs to increase and create their social media presence. However, little is known about the applicant's perceptions of distributed social media content. The study examined applicant perceptions of the social media presence of orthopedic surgery residency programs. We discovered that respondents were most likely to use Instagram to interact with a residency program. Respondents preferred posts including resident biographies, social gatherings/family life, day in the life, program culture, and attending biographies every week. Respondents found some pages that posted inappropriate content, such as drinking, politics, and sexist language, but no respondents reported a decreased likelihood of pursuing orthopedic surgery due to social media content. With the relative lack of diversity in orthopedic surgery as compared to other medical specialties, social media presents the opportunity to influence the future demographics of the specialty. Programs should avoid posting controversial or inappropriate content on social media. Instead, they should highlight the people and educational opportunities within the program. This study provides information to guide residency programs across any specialty using social media to bolster their influence on a broader, more diverse audience of applicants.

## Appendices

Appendix 

**Table 3:** Questionnaire Outline

Question	Response Option 1	Response Option 2	Response Option 3	Response Option 4	Response Option 5	Response Option 6	Response Option 7	Response Option 8	Response Option 9
1. Age	<20	21-34	35 or >						
2. Gender	Male	Female	Nonbinary/Prefer not to say						
3. Sexuality	Heterosexual	Homosexual	Bisexual	Other					
4. Race/ Ethnicity	Asian/Pacific Islander	Black or African American	Hispanic or Latino	Native America or Alaskan Native	White or Caucasian	Multiracial or Biracial	A race/ethnicity not listed here		
4a. If your race/ ethnicity was not listed above, please deacribe it here									
5. Year of Medical School graduation (YYYY)									
6. State of medical school									
7.Are you a current resident	Yes	No							
7a.If yes, what state is the residency program in?									
7b. If yes, what year of residency are you in	1	2	3	4					
8. What is the number of times you applied to an Ortho Residency	1	2	3	4	5 or more				
9. Did/Will you take time off between medical school and residency	Yes	No							
10. Marital Status	Single ( never married)	Married	Divorced						
11. Do you have your own social media account(s)	Yes	No							
11a. If yes, which platofrms	Facebook	Twitter	Instagram	TikTok	LinkedIN	Snapchat	YouTube	Reddit	Other
11aa. If you selected "Other" please elaborate:									
11b. If yes, private or public?									
12.Which social media platform(s) do you use the most? (multiple choice social media platforms listed)									
12a. If you selected “Other,” please elaborate									
13. Have you ever felt discriminated against via content on a social media platform?	Yes	No							
13a. If so, on what basis?	Gender	Race/Ethnicity	Sexuality	Age	Other	None			
13aa. If you selected “Other,” please elaborate:									
14. How frequently have you felt discriminated against on social media?	Daily	Once a week	A few times a week	Once a month	Never				
15. How important is social media presence for residency programs?	Very important	Important	Neutral	Less Important	Not Important				
16. How important was social media presence in your decision to pick a program? If still in medical school, how important will social media presence be?	Very important	Important	Neutral	Less Important	Not Important				
17. Which platforms do you use to engage with residency programs? (multiple choice social media platforms listed as in 11a)									
17a. If you selected “Other,” please elaborate:									
18. Did you utilize social media to evaluate residency programs?	Yes	No							
18a. Which social media platform was MOST effective at interesting you in a residency program? (multiple choice social media platforms listed as in 11a)									
18aa. If you selected “Other,” please elaborate:									
18b. Which social media platform was LEAST effective at interesting you in a residency program? (multiple choice social media platforms listed as in 11a)									
18bb. If you selected “Other,” please elaborate:									
19. Did social media accounts encourage, discourage, or have no effect on your interest in orthopaedics?	Encourage	Discourage	No effect						
19a. If social media had an effect on your interest in orthopaedics, please elaborate:									
20. How often have comments/content on orthopaedic social media accounts bothered you, made you feel uncomfortable, and/or offended you?	Never	Seldom	Often						
20a. Do you care to share the comments or content? Please include which platform, text, and/or graphic involved, if recalled.									
21. Have you viewed any content on orthopaedic social media accounts as inappropriate/unprofessional?	Yes	No							
21a. If yes, please elaborate:									
22. What type of content on an orthopaedic resident program’s social media would appeal to you?	Program details (typical cases, volumes, travel time, attending participation, autonomy, size)	Residents	Attendings	Research accomplishments	Social gatherings/Family life	Educational events	Scheduling details	Program culture	Day-in the life of a resident
22a. If you selected “Other,” please elaborate:									
23. How often would you like to see content from an orthopaedics residency program?	Daily	Weekly	Every two weeks	Monthly	Never				
24. Did you engage with content seen on orthopaedic social media accounts?	Yes	No							
24a. If yes, how?	Commented on the content	Liked the content	Shared the content	Other					
24aa. If you selected “Other,” please elaborate:									
24b. If yes, Do you think interacting with social media helped you get into a residency program?	Yes	No							
25. What stereotypes did you see on social media that related to Orthopaedic surgery?	Gender	Race/Ethnicity	Sexuality	Age	Other	None			
25a. If you selected “Other,” please elaborate:									

## References

[1] Mun F, Badin D, Snow M, Harris AB, LaPorte DM, Aiyer AA (2022). AAMC guidance on interviewing for the 2022-2023 residency application cycle: orthopaedic program director perspectives. JB JS Open Access.

[2] Yong TM, Pappas MA, Ray GS, McManus TG, Coe MP (2021). Analyzing the proliferation of social media use among orthopaedic surgery residency programs. JB JS Open Access.

[3] Feroe AG, Only AJ, Murray JC (2024). Use of social media in orthopaedic surgery training and practice: a systematic review. JB JS Open Access.

[4] Malyavko A, Kim Y, Harmon TG (2021). Utility of social media for recruitment by orthopaedic surgery residency programs. JB JS Open Access.

[5] Matalon SA, Kelil T, Hedgire SS (2022). Enhancing residency recruitment through social media. Radiographics.

[6] Geller JS, Massel DH, Rizzo MG, Schwartz E, Milner JE, Donnally Iii CJ (2022). Social media growth of orthopaedic surgery residency programs in response to the COVID-19 pandemic. World J Orthop.

[7] Joo PY, Moran J, Wilhelm C, Ready J, Rubin LE, Grauer JN (2022). Orthopaedic program reputation and association with social media utilization and engagement. J Am Acad Orthop Surg Glob Res Rev.

[8] Abbas MJ, Jildeh TR, Khalil LS, Buckley P, Mumuni SP, Washington KJ, Okoroha KR (2021). Social media use continues to increase among orthopaedic residency programs in the United States. Arthrosc Sports Med Rehabil.

[9] Wang CX, Kale N, Miskimin C, Mulcahey MK (2021). Social media as a tool for engaging medical students interested in orthopaedic surgery. Orthop Rev (Pavia).

[10] Bellam K, Yakkanti R, Amaya A, Qiu MF, Conte B, Aiyer A (2023). Orthopedic surgery residencies: the leap to social media. Orthopedics.

[11] Lum ZC, Dennison S, Le HV, Bayne CO, Lee CA (2024). Trends in orthopaedic surgery workforce diversity: Analyzing changes over time. J Am Acad Orthop Surg Glob Res Rev.

[12] DelPrete CR, Gianakos A, LaPorte D, Ierulli VK, Mulcahey MK (2023). Perception and usage of social media among women in orthopaedics. J Am Acad Orthop Surg Glob Res Rev.

[13] Bhayani RK, Fick L, Dillman D, Jardine DA, Oxentenko AS, O'Glasser A (2020). Twelve tips for utilizing residency program social media accounts for modified residency recruitment. MedEdPublish (2016).

[14] Steele TN, Galarza-Paez L, Aguilo-Seara G, David LR (2021). Social media impact in the match: a survey of current trends in the United States. Arch Plast Surg.

[15] Butler A, Berke C, Zareef U (2022). Social media and the orthopaedic surgery residency application process. Cureus.

[16] Wang KY, Puvanesarajah V, Suresh KV, Xu AL, Ficke JR, LaPorte D, Kebaish KM (2023). Social media presence is associated with diversity and application volume for orthopedic surgery residency programs. Orthopedics.

[17] Irwin TJ, Riesel JN, Amador RO, Helliwell LA, Lin SJ, Eberlin KR (2021). The impact of social media on plastic surgery residency applicants. Ann Plast Surg.

[18] Brotherton SE, Etzel SI (2023). Graduate medical education, 2022-2023. JAMA.

[19] Lavorgna TR, Gupta S, Maginnis C, Saraf SM, Stamm MA, Wong SE, Mulcahey MK (2023). Persistent lack of female orthopaedic sports medicine fellows. Arthrosc Sports Med Rehabil.

[20] Stevens CR, Merk K, Ierulli VK, Mulcahey MK (2023). Analysis of social media posts that promote women surgeons. J Surg Educ.

[21] Ramirez RN, Franklin CC (2019). Racial diversity in orthopedic surgery. Orthop Clin North Am.

